# Bioinformatics analysis of structures and ligand-bindings of predicted zymogen granule protein observed on Bali cattle (*Bos javanicus*) saliva

**DOI:** 10.5455/javar.2021.h506

**Published:** 2021-06-18

**Authors:** Djoko Kisworo, Sulaiman Ngongu Depamede

**Affiliations:** Faculty of Animal Science, University of Mataram, Mataram, Indonesia

**Keywords:** Mannose-binding lectin, innate immunity, signal peptide, amino acid, sequence, MALDI-TOF/TOF-MS

## Abstract

**Objective:**

Previously, we have shown that predicted zymogen granule protein 16 homolog B (P-G3MZ19) existed in Bali cattle (*Bos javanicus*) saliva. It was suggested that P-G3MZ19 is a member of the mannose-binding lectin family that plays an essential role in innate immunity. In the present study, we aimed to analyze the structure and ligand-binding of P-3MZ19 in Bali cattle saliva.

**Materials and Methods:**

Saliva of four adult healthy Bali cattle was collected, lyophilized, and subjected to two-dimensional (2-D) gel electrophoresis. The target spot of around 17 kDa related to P-G3MZ19 was excised for matrix-assisted laser desorption ionization time-of-flight mass spectrometer/time-of-flight mass spectrometer mass spectrometry analysis and sequencing. The structure and the ligand-binding of P-3MZ19 were analyzed using bioinformatics software programs published elsewhere.

**Results:**

Based on Iterative Threading ASSEmbly Refinement the 3D model of P-G3MZ19 was suggested to have similarities to exo-alpha-sialidase (EC 3.2.1.18); while its ligand-binding sites consisted of seven residues, i.e., 25aa-26aa (Gly-Gly), 95aa (Phe), 138aa (Tyr), 140aa (Leu), 141aa (Gly), and 143aa (Thr).

**Conclusion:**

The structure of P-G3MZ19 of Bali cattle saliva and its ligand-binding sites have been successfully determined by using bioinformatics techniques. The biological and immunological roles of the peptide are currently under investigation based on P-G3MZ19 synthetic peptides.

## Introduction

Zymogen granule is a secretory organelle found in the acinar cells of the pancreas that are responsible for the storage, transport, and secretion of digestive enzymes [1,2]. Chen et al. [3] mentioned that zymogen granule membrane (ZGM) is urgently needed and plays an essential role in the activities of molecular transport machinery in living things, such as sorting, granular channeling, and exocytosis of digestive enzymes. Furthermore, as a post-Golgi transport intermediate, it has been suggested that ZGM contains a variety of proteins involved in exocytosis and granule biogenesis in the exocrine pancreas [4]. Some ZGM proteins have been found to protect pancreatitis [5], regulation of immune cells, and tumorigenesis [6,7]. Several studies have suggested that ZGM, specifically the zymogen granule protein 16, is one of the genes with the most significant downregulation of colorectal cancer tissue [8]. Another study reported that predicted zymogen granule protein 16 (ZGP-16), specifically ZGP-16B, was found in the urine of nude mouse models as a xenograft recipient of human intestinal tumor cells [9]; meanwhile, Lu et al. [10] reported that ZGP-16 B plays a role in breast cancer prognosis.

Based on matrix-assisted laser desorption ionization time-of-flight mass spectrometer/time-of-flight mass spectrometer mass spectrometry (MALDI-TOF/TOF-MS) and Bioinformatics analysis, Depamede [11,12] reported that one of several peptides with a hit identity of G3MZ19 existed in the saliva of Bali cattle (*Bos javanicus*). Although this peptide is yet to be identified, it is considered as the Predicted zymogen granule protein 16 homolog B (P-G3MZ19) or ZGP16 in Taurin (*Bos taurus*) cattle [13]. ZGP-16 is known to be a member of the protein family mannose-binding lectin, which has a significant role in the innate immune system [14,15]. The presence of the ZGP16-like peptide in Bali cattle saliva is quite attractive since it was reported that Bali cattle’s saliva expressed a bactericidal capacity [16]. Whether this is related to ZGP-16, which plays a role in the innate immunity system, still needs to be investigated.

Bali cattle are relatively small in body size (its mature body size is about 600 kg or more) and are indigenous Indonesian beef-type cattle. In Indonesia, Bali cattle still play an important role in meat supply for local consumption and favored smallholders in rural areas due to their adaptability and ease of handling [17,18]. Understanding the biology and physiology of Bali cattle in detail would be very helpful for Indonesia and its neighboring tropical countries, where Bali cattle are also encountered. For those reasons, one of the efforts through performing analysis of structures and ligand-bindings of predicted zymogen granule protein of Bali cattle saliva was conducted. Furthermore, attempts to reveal essential compounds in cattle bodies associated with their physiological functions, using saliva, will be highly significant. It will help develop noninvasive methods that will reduce animal suffering and stress, which are recently becoming a concern for animal welfare. As far as our concerns, this study is the first to analyze the structure of the P-G3MZ19 protein of Bali cattle saliva by using bioinformatics techniques.

## Materials and Methods

### Saliva and two-dimensional (2-D) gel collection and salivary protein analysis using MALDI-TOF-TOF

The saliva of four adult healthy Bali cattle was collected, pooled, and treated according to Depamede [11], with certain modifications. The saliva was collected from cows raised in the experimental pens of the Faculty of Animal Husbandry, University of Mataram. The saliva collection process was carried out noninvasively by trained personnel using a soft, disposable plastic pipette for each individual cow and in accordance with livestock handling regulations in our department. Saliva was lyophilized and subjected to 2-D gel electrophoresis. The sample was dissolved in 150 μl of water, and the protein concentration (5.0 mg/ml) was determined using the BCA assay [19]. The sample was then micro-dialyzed overnight against 5 mm Tris pH 6.8 using 6–8,000 mwco membranes at 5°C, then was lyophilized again and re-dissolved to 4 mg/ml in SDS boiling buffer diluted 1:1 with urea sample buffer before loading.

The 2-D electrophoresis was performed according to the carrier ampholine method of isoelectric focusing [20,21] by conducting isoelectric focusing for 9,600 volt-hrs in a 2.3 mm inner diameter glass tube using 2% pH 3–10 isodalt Servalytes (Serva, Heidelberg, Germany). The sample was added with 1 μg of an IEF internal standard, tropomyosin. This protein migrates as a doublet with a lower polypeptide spot of MW 33,000 and pI 5.2. The enclosed tube gel pH gradient plot for this set of Servalytes was determined with a surface pH electrode.

After equilibration for 10 min in Buffer “O” (10% glycerol, 50 mm dithiothreitol, 2.3% SDS, and 0.0625 M tris, pH 6.8), each tube gel was sealed to the top of a stacking gel that overlaid a 12% acrylamide slab gel (0.75 mm thick). SDS slab gel electrophoresis was carried out for about 4 h at 15 mA/gel. The following proteins (Sigma Chemical Co., St. Louis, MO) were used as molecular weight standards: myosin (220,000), phosphorylase A (94,000), catalase (60,000), actin (43,000), carbonic anhydrase (29,000), and lysozyme (14,000).

### Bioinformatics

In general, bioinformatics techniques used in this study were carried out based on Liu et al. [22], with some modifications to suit the needs. The signal peptide of P-G3MZ19-homologue was predicted by a web-based tool of http://www.cbs.dtu.dk/service/SignalP/, while the subcellular localization was predicted using http://psort.hgc.jp/form.html. The hydrophilic prediction was predicted at http://www.expasy.org/cgi-bin/protscale.pl. Furthermore, the transmembrane domain was predicted through http://www.cbs.dtu.dk/services/TMHMM-2.0/, and the secondary structures were constructed using the software PSIPRED v3.0 (http://bioinf.cs.ucl.ac.uk/psipred/). Finally, the 3D models of G3MZ19-homologue were predicted by I-TASSER (Iterative Threading ASSEmbly Refinement) on the website http://zhanglab.ccmb.med.umich.edu/I-TASSER/

## Results

### 2D gel and peptide analysis of Bali cattle salivary proteins

**Figure 1.**
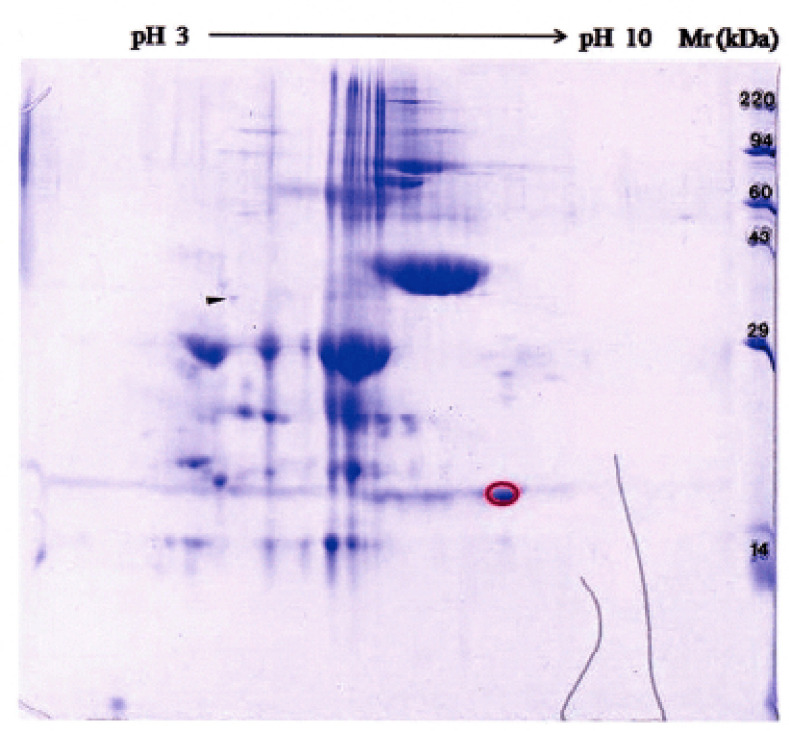
The 2-D gel of Bali cattle salivary proteins. The spot (red circle) was excised and identified by MALDI-TOF/TOF.MS and characterized as P-G3MZ19-homologue.

The result of the 2-D gel of Bali cattle salivary proteins is presented in Figure 1. The relative molecular weight (Mr) standards appear along the basic edge of the acrylamide slab gel. The target spot of around 17 kDa (red circle) was excised for MALDI-TOF/TOF-MS analysis and sequenced according to Depamede [11].

**Figure 2.**
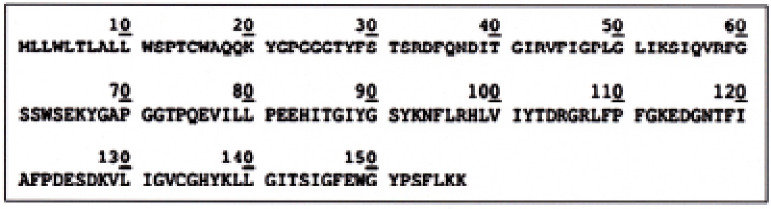
Amino acid residues of Bali cattle P-G3MZ19.

The peptide sequence of 157 residues (Fig. 2) was found to be assigned as P-G3MZ19 homologue (Accession no. gi|296473588|DAA15703.1; gi|359079571|XP_002697975.2), which reinforces previous findings [11,12].

### Hydrophobicity, signal peptide, subcellular localization, and transmembrane domain

**Figure 3.**
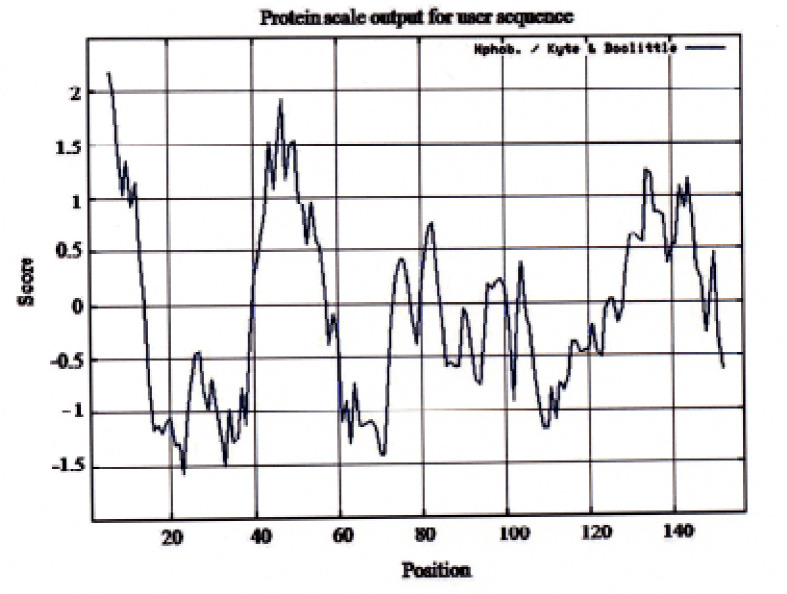
Kyte-Doolittle hydrophobicity profile as a function of sequence position for Bali cattle P-G3MZ19-homologue protein.

The result of the hydrophobicity analysis based on Kyte and Doolittle [23] of Bali cattle P-G3MZ19-homologue is presented in Figure 3. Areas below score 0 indicate the position of the exterior peptides, while the upper section indicates the internal [23].

Furthermore, Keyte and Doolittle [20] also made hydrophobicity of the amino acid scale, with a score of 4.6 for the most hydrophobic amino acids (e.g.isoleusine, I, Ile), and −4.6 for the most hydrophilic (e.g., arginine, R, Arg). From Figure 3, the sharp drops indicate that the exterior parts are in their hydrophilic profile, i.e., Tyr, Glu, and Pro. Meanwhile, the sharp peaks suggest that the interior exists in its hydrophobic residues, i.e., Iso, Val, and Phe. From Figure 3, the exterior parts of the sharp drop shapes are in their hydrophilic profile, i.e., Tyr, Glu, and Pro. Meanwhile, in the interior of the sharp peak existed in its hydrophobic residues, i.e., Iso, Val, and Phe.

### Prediction of signal peptide

**Figure 4.**
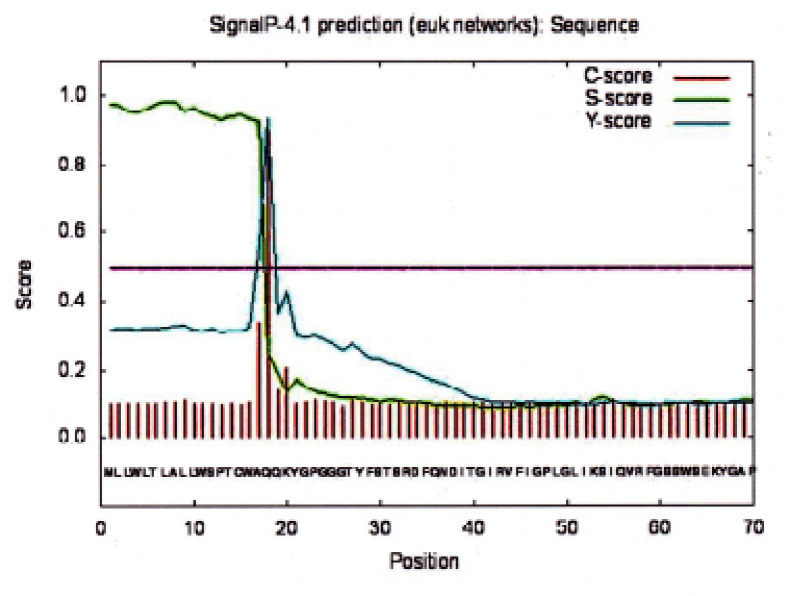
Prediction of P-4.1. Signal peptide of Bali cattle P-G3MZ19-homologue.

The P-4.1 prediction result of the peptide signal of Bali cattle P-G3MZ19-homologue is presented in Figure 4. From Figure 4, it appears that the cleavage site of P-G3MZ19 protein lies between the residues at positions 17 and 18, i.e., CWA-QQ. By employing the Kyte and Doolittle scale [23], we know that the cleavage site is in the hydrophilic position.

### Secondary structure of Bali cattle P-G3MZ19-homolog

**Figure 5.**
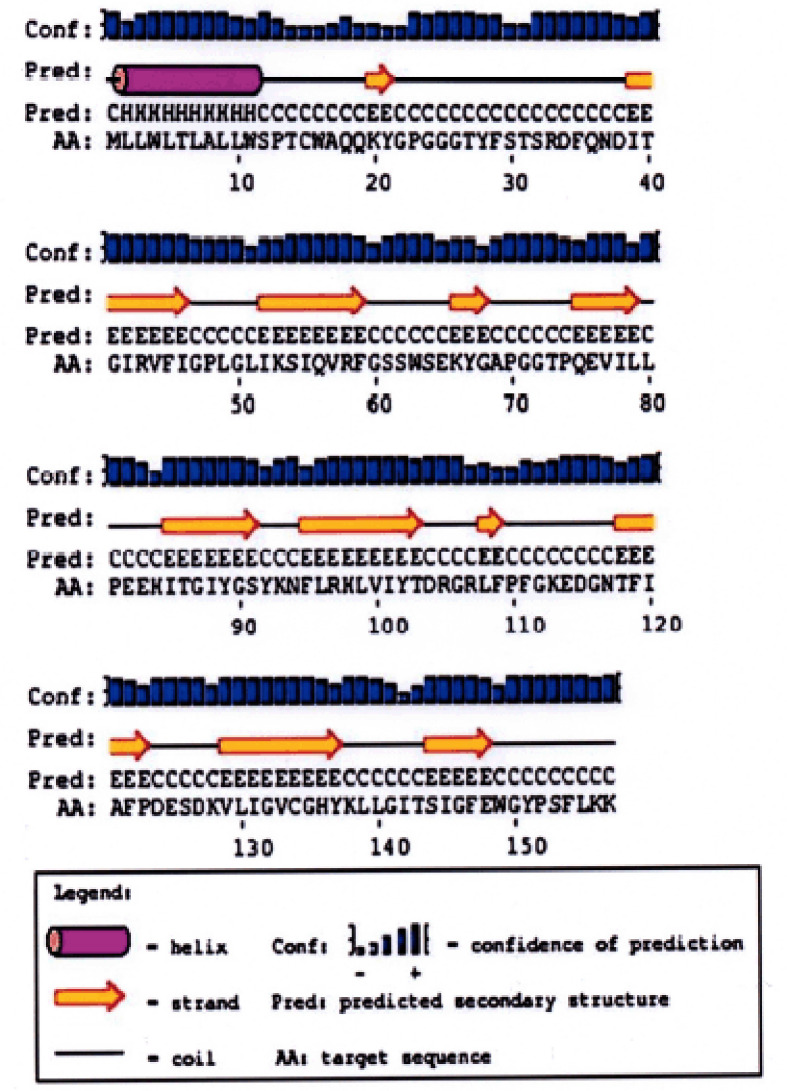
The predicted secondary structure of Bali cattle P-3MZ19.

The predicted secondary pattern of Bali cattle P-G3MZ19 is presented in Figure 5. Only one alpha-helix, 11 beta-strands, and 11 coils were determined in the Bali cattle P-G3MZ19.

### 3D model and enzyme activity predicting

**Figure 6.**
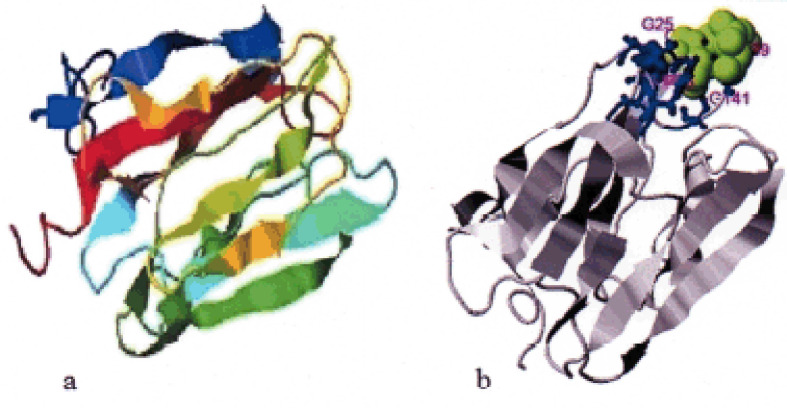
The 3D predicted model (a) and predicted ligand binding sites (b) of Bali cattle P-G3MZ19 based on I-TASSER, chosen from the top five models with the most confidence C-score.

Predicting the activity of an enzyme in the form of a 3D model is essential because it allows a computational approach to be made and makes it easier to examine the structural motifs of some critical evolutionary residues. Furthermore, geometric motifs and similarities and evolution in other protein structures will make decision-making more effective and efficient. The 3D predicted model and predicted ligand-binding sites of Bali cattle P-G3MZ19 based on I-TASSER, are presented in Figure 6a and b, respectively.

## Discussion

The ZGP-16 homolog or P-G3M19 of Bali cattle has been reported by Depamede [16]. Zymogen granule protein-16 homolog-B (ZG16B) was also found out of the 645 seminal plasma proteins in the semen of Indian indigenous MalnadGidda (*Bos indicus*) cattle, based on deep proteome profiling [24]. However, the detailed functions of the ZGP-16, both in Bali cattle and MalnadGidda cattle, have not been revealed yet.

In line with the previous studies, we hypothesized that the presence of P-G3MZ19 in the saliva of Bali cattle might have significant biological potential, so it needs to be revealed. In this study, we can analyze the structures and ligand-bindings of P-G3MZ19 based on bioinformatics means. The 2D SDS-PAGE analysis revealed a spot (Fig. 1) related to molecular weights around 16–19 kDa, which was identified as the P-G3MZ19-homologue, consistent with our previous results [16]. It was also reported that Bali cattle P-G3M19 belongs to the Jacalin lectin family [16], which is similar to the pancreatic ZGM protein 2, the most common granule protein membrane in the granules of pancreatic acinar cells [4]. Lectins are carbohydrate-binding proteins other than enzymes or antibodies. In animals, lectins play essential roles in cell adhesion, the maintenance of membrane polarization, the recognition of pathogens, glycoprotein synthesis, and various protein trafficking or sorting processes [25]. Recently, the lectin pattern for the uterus and placenta of water buffaloes (*Bubalis bubalis*) has been used to study the failure of embryo implantation of *B. bubalis* and *B. taurus *[26]. Previously, it was reported that the pattern of lectin binding could also be used to assess the cattle placenta infected by *Neospora caninum *[27]. Specific studies related to the role of lectin binding pattern in the Bali cattle saliva are still minimal; the most likely approach is to propose its role as a part of the innate immune system in the oral cavity [11].

The hydrophobic profile of P-G3MZ of Bali cattle saliva showed the sharpest drop in its hydrophobic profile in the exterior domain of the protein (Fig. 3). This is in line with the results of the Signal P-4.1 prediction showing a peak fraction at 18 amino acid residue Q (glutamine) with a Kyte-Doolittle score of −3.5 (Fig. 4). Currently, there is a lack of information available with respect to the hydrophobic profile of P-G3MZ of Bali cattle saliva. Since Signal P-4.1 is a program to discover the existence and location of signal peptides and the corresponding cleavage sites in protein sequences rather than the subcellular location itself, it can be suggested that P-G3MZ had a cleavable signal peptide around residues 1–17 with conceivable cleavable sites between 18aa and 20aa.

Biological function depends on the polypeptide structure; in this study, the tertiary structure of Bali cattle P-G3MZ19 contains a single α-helical segment, 11 stranded anti-parallel β-sheet, and 11 motif-forming coils. Based on I-TASSER the 3D model of P-G3MZ19 was predicted to have similarities to an enzyme with the EC number 3.2.1.18 named exo-alpha-sialidase, which also has alternative names alpha-neuraminidase, N-acylneuraminateglycohydrolase, neuraminidase, sialidase. While the predicted ligand-binding sites of Bali cattle P-G3MZ19 consisted of seven ligand-binding site residues, i.e., 25aa- 26aa (Gly-Gly), 95aa (Phe), 138aa (Tyr), 140aa (Leu), 141aa (Gly), 143aa (Thr), the ligand is classified as SUC or sucrose. The presence of SUC ligand in Bali cattle saliva is an exciting subject to be explored. This is mainly related to livestock health management through a glycosylation-modified drug delivery system by utilizing sugar ligand-molecules [28].

It is quite an unexpected result that the 3D model of P-G3MZ isolated from Bali cattle saliva revealed a protein related to exo-alpha-sialidase. It might be possible that the enzyme disembarked from bacteria in the oral cavity of the cattle. Some oral bacteria, including some pathogenic bacteria, convey sialidase to degrade the sialoglycoprotein substrate. They use sialic acid as sugar to increase their growth [29]. Certain bacteria have a gene engine involved in the metabolism of sialic acid, but several other bacteria use an alternative pathway to the sialic acid metabolism. It was also revealed that in a micro bio environment with high mucin content, such as saliva, sialidase plays an essential role in bacterial virulence and pathogenesis [30]. Our previous study showed that Bali cattle saliva was able to suppress the growth of cultured bacteria [16], which might differ from the effect of the existing sialidase. Putting together, as a whole, this shows the complexity of the Bali cattle saliva that needs to be explored further. Similarly, whether the three-dimensional and the ligand-binding patterns revealed in this study are related to P-G3MZ19 peptide activity still need to be explored. 

## Conclusion

Based on I-TASSER, the 3D model of P-G3MZ19 was suggested to have similarities to exo-alpha-sialidase (EC 3.2.1.18). At the same time, its ligand-binding sites consisted of seven residues, i.e., 25aa- 26aa (Gly-Gly), 95aa (Phe), 138aa (Tyr), 140aa (Leu), 141aa (Gly), and 143aa (Thr). As far as our concern, this study is the first to analyze the structure of P-G3MZ19 of Bali cattle saliva by using bioinformatics techniques. The biological and immunological role of the peptide is currently under investigation based on the P-G3MZ19 synthetic peptide.

## List of abbreviations

I-TASSER: Iterative Threading ASSEmbly Refinement; MALDI-TOF/TOF-MS: matrix-assisted laser desorption ionization time-of-flight mass spectrometer/time-of-flight mass spectrometer mass spectrometry; P-G3MZ19: predicted zymogen granule protein 16 homolog B; ZGM: zymogen granule membrane; SDS-PAGE: sodium dodecyl sulphate–polyacrylamide gel electrophoresis.
